# iNaturalist is an open science resource for ecological genomics by enabling rapid and tractable records of initial observations of sequenced biological samples

**DOI:** 10.1098/rsbl.2023.0251

**Published:** 2023-10-04

**Authors:** Jay Keche Goldberg

**Affiliations:** Department of Ecology and Evolutionary Biology, University of Arizona, Tucson, AZ 85721, USA

**Keywords:** genomics, iNaturalist, open science, natural history

## Abstract

The rapidly growing body of publicly available sequencing data for rare species and/or wild-caught samples is accelerating the need for detailed records of the samples used to generate datasets. Many already published datasets are unlikely to ever be reused, not due to problems with the data themselves, but due to their questionable or unverifiable origins. In this paper, I present iNaturalist—a pre-existing citizen science platform that allows people to post photo observations of organisms in nature—as a tool that allows genomics researchers to rapidly publish observations of samples used to generate sequencing datasets. This practice aligns with the values of the open science movement, and I also discuss how iNaturalist, along with other online resources, can be used to create an open genomics pipeline that enables future replication studies and ensures the value of genomics datasets to future research.

## 1. Introduction

**Figure 1.**
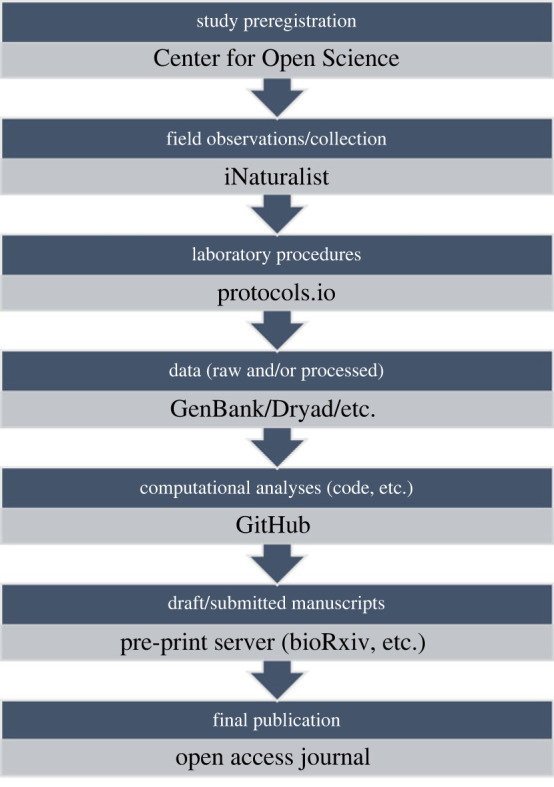
A flowchart outlining an example ‘open genomics pipeline’ with seven key steps and their corresponding open science platform. The second step in this pipeline, publicly recording the initial field observations/collection associated with a study, is the aspect that iNaturalist fulfils. The precise steps, and platforms used to carry them out, necessary for the best open science practices will vary, given the wealth of system-specific databases such as FlyBase or the Sol Genomics Network.

The number of high-quality published genomes has increased rapidly in recent years [1], and the feasibility of sequencing multiple individuals of species with large heterozygous genomes has enabled pan-genomics with eukaryotic organisms [2]. Once restricted to prokaryotes with small genomes [3], there are now several plant and animal species with publicly available pangenome databases [4,5]. Evolutionary biologists are routinely using whole-genome sequencing to observe responses to climate change [6] and experimental manipulation [7] in real time. Many laboratories and consortia are publishing genomes as fast as possible to make them available to the broader scientific community [8], but often publish their data in minimalist reports [9] that sometimes lack even basic descriptions of the data itself [10]. The explosion of genomic data, while scientifically exciting, presents a dilemma if details regarding the collection of source sample(s) are not properly recorded and made available to the broader scientific community. Datasets originating from wild samples require more rigorous documentation of the originating samples to ensure their long-term value—especially when they are rare or cryptic species, or members of poorly resolved clades. Current best practice is to submit voucher specimens to museums/herbaria, but many researchers fail to do so and when they do the degradation of preserved samples can create issues for later validation, as natural pigmentation fades over time or fine-scale structures important for identification are inadvertently damaged during transport or long-term storage. Travelling to consult collections in person is also difficult or impossible for many researchers. Many museums have begun digitizing their collections to alleviate this burden and make their specimens open access, but this practice is not yet universal and requires resources that are unavailable to underfunded institutions [11]. The ethics of collecting samples from natural populations are hotly debated, considering widespread ecological degradation [12], and it is of critical importance that biologists minimize the environmental impact of their research. When extra samples for museum deposition cannot be collected due to ethical concerns, it creates a significant gap for open genomics research. iNaturalist—a platform where users post observations of wildlife and experts identify them—could be a valuable tool for researchers who wish to improve the reusability of their data while minimizing the environmental impact of sample collection. Observations posted on iNaturalist can represent the whole organism in cases where a small non-lethal sample is sufficient for sequencing studies, and the precise individual sampled in cases where an entire organism is required, thereby eliminating the need for additional sampling for record-keeping purposes. Furthermore, the publicly accessible nature of iNaturalist observations (one can access them without an account on the platform) makes it ideal for tackling the lack of robust, easily accessible, information regarding the originating samples used to generate publicly available sequencing datasets—and help create a fully open genomics data pipeline (figure 1). This practice is not mutually exclusive with the use of formally curated museum specimens—especially when there are no ethical concerns surrounding the collection of study species—and can be used in combination with established practices to expand the availability of information surrounding sample/specimen collection.

## 2. What is iNaturalist?

iNaturalist is a citizen science platform that allows users to upload photos from an internet-connected device (smartphone, computer, etc.). It is not the first or only citizen science platform to accomplish this—many region-specific databases also exist—but its global scope and large user-base makes it the best suited for use in genomics research. Knowledgeable identifiers, often actively publishing researchers or museum curators, identify observations added to the database. These photo observations are also accompanied by metadata—the date/time and location at which the photo was taken—and sometimes include specific notes regarding the sex, life stage, etc. of the observed organism (these are often filled in by identifiers). Any discussion of the observations by the observer and identifiers is also recorded and associated with it. iNaturalist has already proven its value to ecologists and provided data for studies regarding invasion dynamics [13] and animal behaviour [14].

## 3. An open genomics pipeline

Open-access journals have become commonplace and many funding agencies mandate that results be published in them. Public repositories for various forms of data (GenBank, Dryad, etc.)—and the code needed to analyse them (Github)—exist and are often free to contribute to. Some model species and popular study clades even have their own dedicated repositories (e.g. Flybase, Sol Genomics Network). Resources for publishing step-by-step methodologies (protocols.io) also exist. Yet, until the advent of iNaturalist (and other citizen science platforms), there was no way to freely publish open-access natural history observations other than within peer-reviewed publications. Now, however, it is possible to instantly upload photos from the field, have them automatically associated with key metadata (time and location), and make them freely available to both the scientific community and the broader public using iNaturalist. This makes it a valuable tool for ecological and evolutionary geneticists to improve their data pipelines and better align with open science practices.

iNaturalist's utility lies in how it allows researchers to associate publications with field observations via their unique URLs (example user profile and observation can be found in Web resources) that provide an easy-to-follow paper trail. This allows future researchers to verify the identity of the initial sample and collection details. This is critical for species that are likely to have their taxonomy revised as their identity can be followed through disagreements between systematists based on their observable traits. The iNaturalist taxon framework generally follows the Catalogue of Life but is manually updated by a global team of curators, many of whom are also curators of physical herbarium/museum collections and formally trained taxonomists. Knowledgeable users can flag species or taxa for curation and the platform records these notes, alongside curators' responses and/or changes. This detailed digital paper trail allows for minor identification errors (e.g. those that do not meaningfully alter the outcome of a study) or post-publication taxonomic revisions to be recorded and linked to the final dataset and/or publication without the need for formal corrections.

To maximize the utility of iNaturalist for producing digital vouchers, researchers should provide as much detail as possible when submitting observations. At a bare minimum, all metadata fields (location, date/time, life stage, sex, etc.) should be completed. Multiple clear and descriptive photographs showing any/all traits necessary for identification should be submitted. When necessary, microscopy images of fine-scale morphology to aid with expert identification should be submitted. Depending on the study in question, further details (text annotations and/or photographic evidence) regarding local habitat or environmental conditions should also be provided; this information could be valuable for interpreting the outcomes of transcriptomic or population genetic studies examining organismal responses to local environments or rapid anthropogenic change. If observed samples are submitted to physical museum/herbarium collections, the voucher code and information about the specimen should also be provided in the notes section. If/when sequencing data are available, database information (e.g. GenBank accession numbers) should be provided. Researchers could also describe the purpose of sample collection (experimental design, extraction procedure, etc.), but it may be preferable to record this information with a hypothesis registry service instead. Ultimately, iNaturalist observations for research purposes should include all the information necessary for the scientific community to validate and replicate study findings.

When accessed in bulk through the Global Biodiversity Information Facility (GBIF), sets of iNaturalist observations can be given digital object identifiers (DOIs) that enable replication studies [15,16], and, within the iNaturalist platform, observations can be collected into projects. Since it is now common to find genomics studies that include hundreds or thousands of samples collected from multiple species across broad geographical or long temporal scales [17,18], the collation of collection records into tractable projects/datasets will enable researchers to keep track of the samples used in a study that they may be planning, carrying out, or have already published. Any projects that an observation is a part of are shown underneath the observation, thus making it easy to track how researchers have used, or are planning to use, a sample/dataset. In addition to tracking important metadata regarding the use of scientific samples for open and repeatable science, this gives the public deeper insight into the science of the species they see in daily life and a direct line to the researchers conducting it.

## 4. Future directions

While it is a powerful tool, iNaturalist is not perfect. Like all centralized services there is a risk of data loss should their infrastructure be compromised by natural disaster, malicious actors or financial setbacks. Much like private data storage, all important resources should be backed up and archived in other trusted databases. This could be accomplished by depositing datasets in other locations, be it a system-specific repository, regional database, or general-purpose repository (e.g. Zenodo). This process could likely be automated using computational tools that access iNaturalist via their application programming interface (API). Their API could also be used to automate the process of bulk observation uploads and/or modifying their descriptions to include links to resulting datasets (e.g. GenBank submissions) as they become available. API use is currently subject to strict rate limits (100 requests per minute; 5 GB per hour), which could prove to be a bottleneck for large high-throughput studies, but this will likely increase as they continue to develop and improve their digital infrastructure. It is also important to consider how iNaturalist observations will be referenced in other databases, ideally they should be referenced reciprocally such that observations reference subsequent datasets and these datasets reference back to the initial observations. Ultimately, propagating and eventually standardizing this process will require further discussion about and development of data management practices, but iNaturalist in its current form is already a valuable tool for creating open ecological genomics research.

## 5. Conclusion

As the genomics revolution continues to open doors to research on the ecology and evolution of previously impossible-to-study species, the need for better documentation of data origins will increase dramatically. While online photo observations are not a full-fledged replacement for formally curated museum specimens, iNaturalist is a platform that researchers can use to rapidly publish field observations of samples that are eventually used in sequencing projects. When combined with other open science resources, it creates an open genomics data pipeline that allows both the scientific community and public-at-large to have better insight into the process behind genomics research.

## 6. Web resources

*iNaturalist*

Homepage: https://www.inaturalist.org/

GBIF homepage: https://www.gbif.org/

iNaturalist user profile: https://www.inaturalist.org/people/6089000

Example observation: https://www.inaturalist.org/observations/134334492

*Public information repositories*

protocols.io: https://www.protocols.io/

Dryad: https://datadryad.org/stash

Github: https://github.com/

GenBank: https://www.ncbi.nlm.nih.gov/genbank/

European Nucleotide Archive (ENA): https://www.ebi.ac.uk/ena/browser/home

FlyBase: https://flybase.org/

WormBase: https://wormbase.org/

The Arabidopsis Information Resource (TAIR): https://www.arabidopsis.org/

Sol Genomics Network: https://solgenomics.net/

Saccharomyces Genome Database: https://www.yeastgenome.org/

Catalogue of Life: https://www.catalogueoflife.org/

Center for Open Science Preregistration Portal: https://www.cos.io/initiatives/prereg

International Nucleotide Sequence Database Collaboration: https://www.insdc.org/

*Biology focused pre-print servers*

bioRxiv: https://www.biorxiv.org/

EcoEvoRxiv: https://ecoevorxiv.org/

medRxiv: https://www.medrxiv.org/

Zenodo: https://zenodo.org/

## Data Availability

This article has no additional data.
